# Enhancement sensitivity of TRPV1 in dorsal root ganglia via the SP-NK-1 pathway contributes to increased bladder organ sensitivity caused by prostatitis

**DOI:** 10.3389/fnins.2024.1484980

**Published:** 2024-10-31

**Authors:** ZhiPeng Jiang, Wen Luo, Lei Liu, ZongMin Long

**Affiliations:** Third Affiliated Hospital of Zunyi Medical University, First People’s Hospital of Zunyi, Zunyi, China

**Keywords:** prostatitis, transient receptor potential vanilloid 1, cross-organ sensitization, SP-NK-1 pathway, urination disorder

## Abstract

Chronic prostatitis/chronic pelvic pain syndrome is a prevalent condition affecting the male urinary system. The urinary dysfunction resulting from this disorder has a direct or indirect impact on the patient’s quality of life. Recent studies have suggested that organ cross-sensitization between the prostate and bladder may elucidate this phenomenon; however, the specific molecular mechanisms remain unclear. In this study, we simulated the urinary symptoms of prostatitis patients using an animal model and examined the expression of relevant proteins within the prostate-bladder sensitized neural pathway. We found that transient receptor potential vanilloid 1 (TRPV1) protein is highly expressed in the dorsal root ganglia (DRG) that co-innervate both the prostate and bladder, potentially increasing the sensitivity of TRPV1 channels via the substance P-neurokinin 1 (SP-NK-1) pathway, which may exacerbate micturition symptoms. Furthermore, in the absence of bladder inflammation, elevated levels of neurogenic substances in bladder tissue were found to sensitize bladder sensory afferents. Collectively, these results underscore the significant role of TRPV1 in bladder sensitization associated with prostatitis, suggesting that the inhibition of TRPV1 along this sensitization pathway could be a promising approach to treating urinary dysfunction linked to prostatitis in the future.

## 1 Introduction

Prostatitis is a prevalent urinary tract disease in adult men, with an incidence rate ranging from 4.5% to 9% and a recurrence rate of around 50% in elderly patients (Polackwich and Shoskes, 2016). Clinically, prostatitis is categorized into four types, with type III prostatitis (chronic prostatitis(CP)/chronic pelvic pain syndrome(CPPS)) being the most prevalent (Pena et al., 2021). This type primarily presents with pelvic pain and lower urinary tract symptoms (Nickel et al., 1999). The pathogenesis of CP/CPPS is multifactorial, involving immune responses, infections, endocrine influences, neurological factors, and psychological components (Pena et al., 2021). The lower urinary tract symptoms associated with CP/CPPS may stem from both morphological and functional alterations in the lower urinary tract, including bladder outlet dysfunction, bladder neck hyperplasia, and pelvic floor muscle spasms (Hochreiter and Z’Brun, 2004). However, these causes frequently overlap, leading to ineffective treatment strategies for LUTS and the recurrence of symptoms. Consequently, a comprehensive understanding of the pathogenesis of LUTS resulting from prostatitis is essential for advancing management strategies in the future.

Over the past decade, the theory of cross-sensitization between pelvic organs has been established. This theory suggests that painful stimuli from one affected pelvic organ can be transmitted to nearby healthy organs, leading to dysfunction in the healthy organs (Qin et al., 2005; Malykhina, 2007). Previous research has indicated a relationship between the colon and bladder in terms of cross-sensitization (Grundy and Brierley, 2018). However, as the focus shifts towards understanding the development of prostatitis, recent studies have suggested that chemically induced prostatitis models can lead to overactive bladder symptoms without causing inflammation in the bladder (Funahashi et al., 2019; Mizoguchi et al., 2019; Ni et al., 2019; Aydogdu et al., 2021a; Aydogdu et al., 2021b). This observation points towards a potential cross-sensitization between the prostate and bladder.

Transient receptor potential vanilloid 1 (TRPV1), a non-selective cation channel, plays a crucial role in the cross-sensitization of pelvic organs (Pan et al., 2010; Malykhina et al., 2013; Yoshikawa et al., 2015). This channel is widely expressed in both neural and non-neural tissues (Bevan et al., 2014). Activation of TRPV1 under thermal, physical, and chemical stimulation, among other conditions (Aghazadeh Tabrizi et al., 2017), can lead to the generation of intracellular signaling cascades (Caterina et al., 1997; Frias and Merighi, 2016; Satheesh et al., 2016; Maximiano et al., 2024). An animal experimental study showed that changes in prostate inflammation do not depend on the activation of TRPV1 (Roman et al., 2020). Nevertheless, the expression of the nociceptive substance TRPV1 in sensory neurons innervating the prostate consistently increases during prostatic inflammation. Additionally, the lack of TRPV1 channels impedes the development of bladder overactivity (Frias et al., 2012; Kitagawa et al., 2013). Therefore, exploring the role of TPRV1 in chronic prostatitis and revealing the pelvic organ sensitization mechanism will help treat this disease.

In this study, abnormal urinary behavior was induced by injecting formalin into the prostate of rats to mimic the lower urinary tract symptoms of CPPS patients. We examined the expression of TRPV1 protein and related proteins in the prostate-bladder cross-organ cross-sensitization pathway to investigate the pathogenesis of CP/CPPS.

## 2 Materials and methods

### 2.1 Animals and groups

Ten male Sprague–Dawley rats were purchased from Jiangsu Huachuang Sino Pharmaceutical Technology Co., Ltd. Ten 9-week-old SD rats were randomly divided into 2 groups after being conventionally fed for 1 week: the prostatitis group (*n* = 5) and the control group (*n* = 5). The prostatitis group received an injection of 50 ul of 5% formalin (100 ul total) into the ventral lobe of the prostate to induce inflammation, while the control group received an equal volume of normal saline (Funahashi et al., 2019). To prevent leakage of injected drugs, it is recommended to keep the needle at the injection site for approximately 30 seconds and then wipe the site with a cotton swab (Aydogdu et al., 2021b). All rats were sacrificed on day 20 to collect prostate, bladder, and dorsal root ganglion (bilateral L6 to S1) tissues. This study was approved by First People’s Hospital of Zunyi Ethics Committee.

### 2.2 Assessment of urinary behavior by urine spot test

**FIGURE 1:**
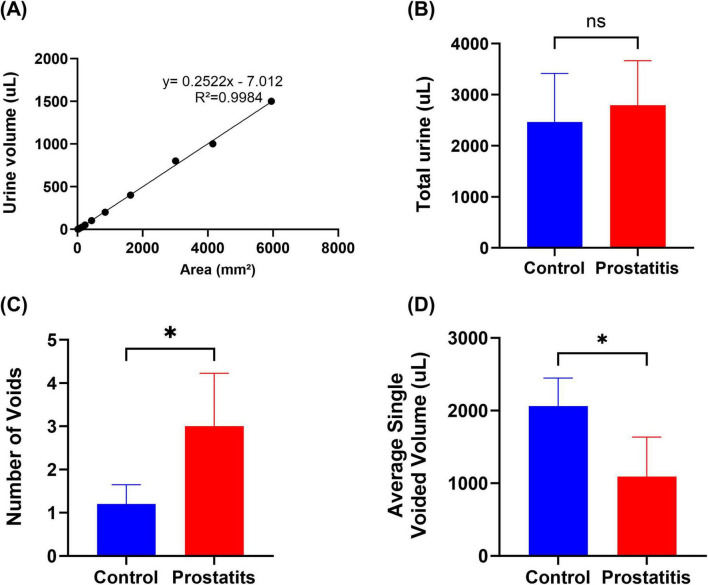
Urination behavior of rats with prostatitis. **(A)** The volume-to-area standard curve with formula. **(B)** Total urine output in 4 h. **(C)** Quantification of the number of micturition spots. **(D)** Average single urine output. Differences between groups were compared by an unpaired *t*-test. Data are expressed as mean ± SEM. *Significant difference compared with the Control group, *p < 0.05; “ns” indicates *p* > 0.05.

Rat urine was collected and dropped onto filter paper in volumes of 1, 5, 10, 25, 50, 100, 200, 400, 800, 1000, and 1500μL to create urine spots. The corresponding spot areas were measured using ImageJ, with each measurement being repeated three times to obtain an average area for each volume. Subsequently, an Excel spreadsheet was utilized to generate a standard curve correlating urine volume to urine spot area (Figure 1A). In the urine spot experiment, the rats were placed in a cage covered with No. 4 filter paper (no autofluorescence) with a thickness of 0.7 mm and allowed to urinate freely for 4 hours on days 0, and 20. Subsequently, collect the urine-stained filter paper, observe it with a UV analyzer, image it under 365 nm UV light, and then use ImageJ analysis to perform statistical analysis on the urine-stained image (Luo et al., 2023).

### 2.3 Histological analyses

All rats were sacrificed after anesthesia, and the prostate and bladder tissues were removed. The excised tissue was fixed in 4% paraformaldehyde for 24 hours and subsequently embedded in paraffin. The prostate and bladder tissues were then sliced into 5um thick sections, stained with hematoxylin-eosin (HE), and examined under a light microscope to assess morphological changes in the prostate and bladder.

### 2.4 Immunofluorescence analysis

The prepared bladder tissue sections were washed with TBST buffer, followed by the addition of blocking serum. Primary antibodies SP and TRPV1(overnight at 4°C) and HRP-conjugated secondary antibodies (37°C for 30 min) were added for incubation, followed by a drop of Cy3 tyramide (37°C for 30 min). Finally, DAPI nuclear staining was performed, and the slides were sealed with a mounting solution containing an anti-fluorescence quench agent. Finally, the images were observed and taken under a fluorescence microscope.

### 2.5 Western blotting analyses

Rat L6-S1 dorsal root ganglion (DRG) and bladder tissues were collected, frozen at −80°C, and homogenized. Proteins were extracted from rat bladder tissue and DRG respectively, and the protein concentration in each tissue was measured using a BCA kit. Subsequently, the extracted protein was mixed with buffer at a ratio of 4:1, added to boiling water, and kept in a boiling water bath for 10 min. Denatured proteins are then loaded onto SDS-PAGE for electrophoresis. After electrophoresis, the protein is transferred to the membrane, and blocking is performed after the membrane transfer is completed. The DRG protein is hybridized with the primary antibody TRPV1 (1:2000) or SP (1:2000) or NK-1 (1:1000) and the secondary antibody (1:1000). The primary antibody TRPV1 (1:2000) is used for bladder tissue protein detection or SP (1:2000) hybridization. Image-pro plus (IPP) software was used to measure the optical density value of the protein band and compare the expression levels of the two groups of proteins.

### 2.6 Statistical analysis

All values were expressed as mean ± SEM and statistical analysis was conducted using an unpaired *t*-test. *p* < 0.05 was considered as a statistically significant difference. All data were analyzed using GraphPad Prism version 10.00.

## 3 Results

### 3.1 Histopathological features of the prostate and bladder after formalin induction

**FIGURE 2:**
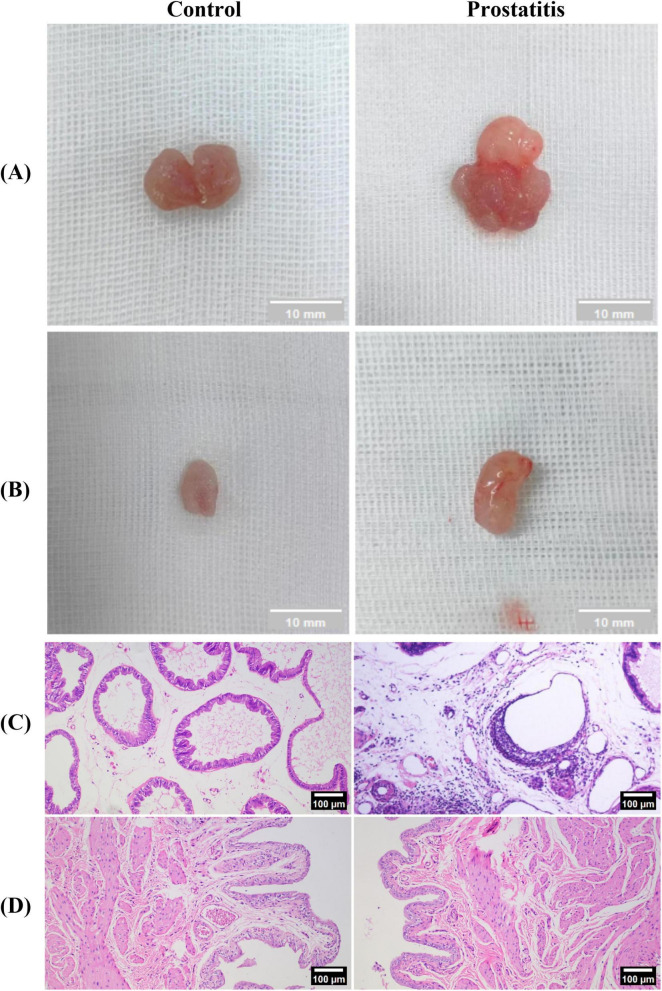
Establishment of the rat model of prostatic inflammation. **(A,B)** General view of prostate tissue and bladder tissue in each group; **(C,D)** HE staining of prostate tissue and bladder tissue in each group (magnification 100 × ); Scale bar, 100 × magnification is 100 um.

We observed that compared with the control group, the prostate surface of rats in the prostatitis group was rougher, and the adhesion to the surrounding tissue was harder and harder (Figure 2A). We speculate that this phenomenon may be attributed to inflammation leading to prostate stroma fibrosis and hyperplasia of prostate tissue. The bladder tissue of both groups exhibited a smooth texture overall, showing no apparent adhesion to the surrounding tissue (Figure 2B). Under light microscopy, we observed varying degrees of damage to the prostate acinar structure in rats from the prostatitis group. This included irregular lumen and disordered arrangement of acinar epithelial cells. Additionally, a significant infiltration of inflammatory cells was noted in the glandular stroma, along with fibrous connective tissue hyperplasia to a certain degree. These changes were not observed in the control group (Figure 2C). For bladder tissue, there was no obvious inflammatory cell infiltration in both groups (Figure 2D).

### 3.2 Prostatitis rats exhibited abnormal voiding behavior

Experimental data demonstrated no significant difference in the total urination volume between the two groups (Figure 1B). However, the prostatic inflammation group exhibited an increase in the number of urinary spots (Figure 1C) and a decrease in single urination volume (Figure 1D). These findings suggest that formalin-induced prostatitis can result in alterations in bladder function that mirror symptoms commonly observed in patients with prostatitis.

### 3.3 Prostatitis rats exhibited increased expression of SP, NK-1, and TRPV1 in L6-S1 DRG

**FIGURE 3:**
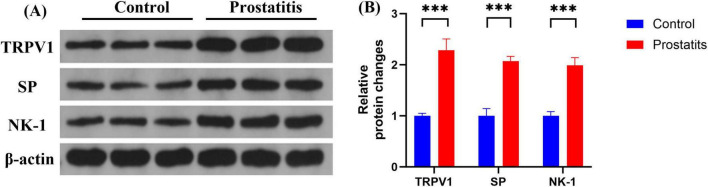
Expression of TRPV1, SP, and NK-1 in DRG. **(A)** Western blotting analysis results show increased expression of TRPV1, SP, and NK-1 in DRG in the prostatitis group. **(B)** The difference is statistically significant compared to the control group. ***p < 0.001 versus the control group. Data represents the mean ± SEM.

Although studies have demonstrated that prostatitis can enhance the expression of TRPV1 in dorsal root ganglia (DRG) and contribute to organ sensitization (Zhang et al., 2019; Wang et al., 2022), there is little research investigating the mechanisms underlying sensitization following the upregulation of TRPV1. SP, a neuropeptide that acts on the NK-1 receptor, is closely associated with the expression and functionality of TRPV1. An increase in the expression of SP and its receptor may result in heightened activity of TRPV1 (Zhang et al., 2007; Tang et al., 2008; Lapointe et al., 2015; Jo et al., 2016). In our study, we aimed to investigate the functional relationship between SP, NK-1, and TRPV1 in the DRG following prostatitis. We observed that rats with prostatitis exhibited increased expression levels of SP, NK-1, and TRPV1 in the L6-S1 DRG. These differences were statistically significant when compared to the control group (Figure 3).

### 3.4 Prostatitis rats exhibited increased expression of SP and TRPV1 in bladder tissue

**FIGURE 4:**
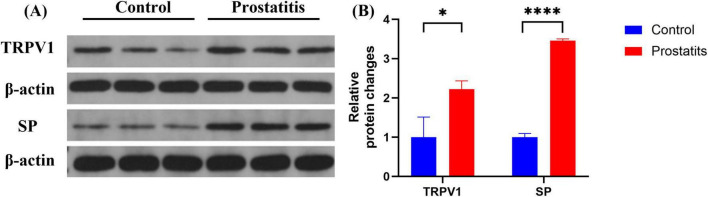
Expression of TRPV1 and SP in bladder. **(A)** Western blotting analysis results show increased expression of TRPV1 and SP in bladder in the prostatitis group. **(B)** The difference is statistically significant compared to the control group. *p < 0.05 versus the control group ****p < 0.0001 versus the control group. Data represents the mean ± SEM.

Organ sensitization plays a significant role in the development of overactive bladder (Grundy and Brierley, 2018). In experimental colitis animal models, an association has been observed between TRPV1 expression and the occurrence of overactive bladder (Asfaw et al., 2011). Therefore, we aimed to investigate the relationship between bladder overactivity induced by prostatitis and the associated changes in relevant substances within the bladder. We observed that prostatitis rats demonstrated elevated expression of SP and TRPV1 in the bladder. These differences were found to be statistically significant when compared to the control group (Figure 4).

### 3.5 Expression and distribution of SP and TRPV1 in bladder tissue

**FIGURE 5:**
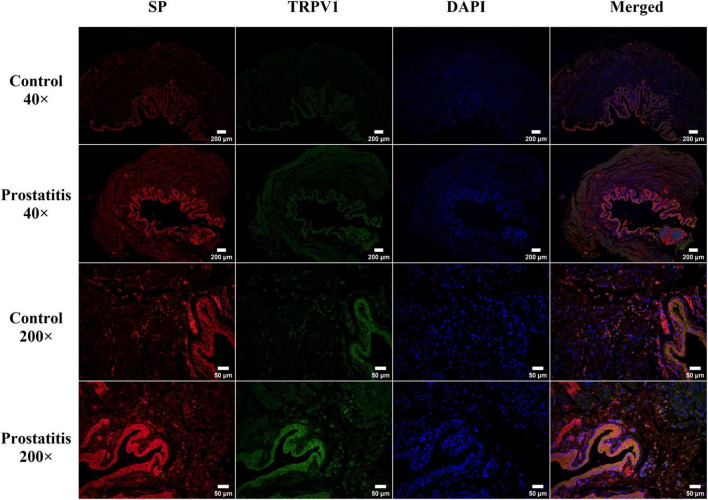
Immunofluorescence staining of transient receptor potential vanilloid 1 (TRPV1; green) and substance P (SP; red) in rat bladder tissue. TRPV1 and SP are predominantly expressed in the bladder urothelium, with a minor expression noted in the bladder muscle layer. Strong expression of both TRPV1 and SP was observed in the bladder urothelium of the prostatitis group. Co-localization analysis indicated that TRPV1 and SP are primarily distributed within the bladder urothelium. Scale bar, 40 × magnification is 200 um; Scale bar, 200 × magnification is 50 um.

Bladder C-fibers play a crucial role in the regulation of micturition reflexes, possessing both afferent and efferent sensory functions (Lazzeri et al., 2009). The aforementioned research results indicate that prostatitis can lead to an increased expression of SP and TRPV1 in the bladder. Notably, TRPV1 is crucial for sensory afferent signaling, while SP is significant for sensory efferent pathways. Consequently, we conducted immunofluorescence to investigate the distribution of the two substances within bladder tissue. We observed that TRPV1 and SP are co-localized and expressed in the bladder urothelium. Following the onset of prostatitis, there is an increase in the fluorescence intensity of both substances in the bladder urothelium (Figure 5).

## 4 Discussion

The findings of this research demonstrate that: (1) Overactive bladder syndrome can result from non-bacterial prostatitis. These symptoms include urinating more frequently and reducing urine volume per voiding episode. (2) Although no inflammatory cell infiltration was observed in the bladder of non-bacterial prostatitis, the expression of TRPV1 and neurogenic SP was increased in the bladder, both of which are co-expressed in the bladder urothelium. (3) Prostatic inflammation can result in sensitization of the SP-NK-1 pathway in capsaicin-sensitive C-fibers, potentially leading to an increase in TRPV1. This suggests that prostatitis may upregulate TRPV1 in DRG via the SP-NK-1 pathway, ultimately causing sensitization of internal organs and worsening bladder overactivity.

Histopathology of the prostate plays a critical role in the assessment of chronic prostatitis model effectiveness. Increased urination frequency is one of the main symptoms in humans and one of the important phenotypic criteria for establishing rodent models. In a clinical study, it was observed that 86% of prostate specimens from patients with chronic nonbacterial prostatitis exhibited chronic inflammatory changes (Doble et al., 1989). In this study, we observed inflammatory changes in the prostate tissue in rats with non-bacterial prostatitis, along with increased frequent urination. These findings suggest the successful establishment of the prostatitis model.

Histopathological results indicated that non-bacterial prostatitis did not exhibit inflammatory cell infiltration in the bladder, but did lead to bladder overactivity. These findings are consistent with the results reported by previous studies (Funahashi et al., 2019; Mizoguchi et al., 2019; Ni et al., 2019; Aydogdu et al., 2021a; Aydogdu et al., 2021b). However, some studies have indicated that non-bacterial prostatitis models do not exhibit alterations in urinary behavior (Yamaguchi et al., 2019). It is hypothesized that this discrepancy could be attributed to different modeling methods. To explain the phenomenon that prostatitis leads to bladder sensitization, some studies suggest that the bipartite DRG innervating the prostate and bladder may be involved in prostate-bladder neural crosstalk (Chen et al., 2010; Funahashi et al., 2019). Chen (Chen et al., 2010) et al. discovered that bladder and prostate afferent neurons converged in the L1-L2 and L6-S1 DRG using retrograde fluorescent labeling techniques. Following bladder denervation, there was a notable decrease in the number of convergent cells. Funahashi et al. (2019) observed that formalin injection into the right ventral lobe of the prostate in rats with right pelvic nerve transection did not result in bladder overactivity. In contrast, injection into the left prostate lobe led to more frequent bladder overactivity. Ni et al. (2019) demonstrated that prostatic inflammation can lead to prolonged hyperexcitability of capsaicin-sensitive C-fiber bladder afferent neurons. Due to the convergence of DRGs in the bladder and prostate, we speculate that the high excitability of convergent DRG neurons after prostatitis may be the process of transmitting noxious stimulation from the prostate to adjacent non-stimulating structures, ultimately resulting in functional alterations in the bladder.

TRPV1 is a cation channel that can be activated by various stimuli and plays a critical role in the pathophysiology of overactive bladder (Juszczak and Thor, 2012). TRPV1 is expressed in primary afferent neurons of unmyelinated, small-diameter sensory nerve fibers as well as in non-nervous tissues. Additionally, TRPV1 expression has been identified in bladder tissue and bladder afferent neurons within the urinary system (Szallasi, 2023). Recent studies have discovered that afferent neurons from the prostate and bladder come together in the L6-S1 DRG, bladder overactivity induced by prostatitis and alterations in TRPV1 in bladder afferent neurons rely on the activation of pelvic nerves (Funahashi et al., 2019). Activating TRPV1 c-fibers triggers the release of neuropeptides such as SP and CGRP from peripheral nerve terminals (Engel et al., 2012; Birder and Kullmann, 2018). These neuropeptides impact the excitability of afferent nerves, resulting in detrusor contraction and neurogenic inflammation (Birder and Kullmann, 2018; Vanneste et al., 2021). In our study, we found that SP and TRPV1 protein levels were increased in bladder tissue after prostatitis and were co-expressed in the bladder urothelium. We speculated that prostatitis may trigger the release of neuropeptide SP by bladder-efferent nerves via the prostate-bladder cross-organ sensitization pathway. Subsequently, SP could potentially interact with TRPV1 at peripheral sensory nerve endings, resulting in sensitization of bladder afferent nerves. Multiple studies have demonstrated that prostatic inflammation upregulates the expression of TRPV1 in DRG (Funahashi et al., 2019; Zhang et al., 2019; Igarashi et al., 2021). Subsequently, TRPV1 facilitates the release of SP via various intracellular signaling pathways (Tang and Nakata, 2008; Yang et al., 2010). SP can subsequently bind to NK-1 receptors and activate PKC, ultimately amplifying the sensitivity of TRPV1 (Zhang et al., 2007; Srinivasan et al., 2008; Lapointe et al., 2015). In our study, we observed increased expressions of SP, NK-1, and trpv1 in the DRG of rats within the prostatitis group. We hypothesized that prostatic inflammation may trigger the activation of TRPV1 channels in the DRG, resulting in the release of SP. This released SP could potentially bind to NK-1 receptors via autocrine or paracrine mechanisms, thereby activating signaling pathways in the DRG, such as the PKC pathway. This sequence of events could potentially heighten TRPV1 sensitivity in the DRG, leading to increased excitability of the DRG and exacerbation of overactive bladder symptoms in patients with prostatitis.

This study has certain limitations. Specifically, we identified neurogenic substances in the bladder, but further in vitro experiments are needed to fully understand the specific mechanism of action of TRPV1 in SP and peripheral nerve terminals. Additionally, while we detected the expression of related proteins in dorsal root ganglion (DRG) in the sensitization of bladder organs caused by prostatitis, future research could utilize the whole-cell clamp recording method to evaluate the electrophysiological properties of DRG under the action of different substances.

## 5 Conclusion

This study suggests that abnormal urinary behavior caused by prostatic inflammation caused by formalin may be related to cross-sensitization between the prostate and bladder. The heightened sensitivity of TRPV1 expression in the DRG via the SP-NK-1 pathway plays a crucial role in this cross-sensitization mechanism. Inhibition of TRPV1 along the sensitization pathway may hold promise as a therapeutic approach for treating urinary dysfunction caused by prostatitis.

## Institutional review board statement

All the protocols and experiments were approved by the Third Affiliated Hospital of Zunyi Medical University.

## Data Availability

The raw data supporting the conclusions of this article will be made available by the authors, without undue reservation.
